# Prefrontal contributions to the stability and variability of thought and conscious experience

**DOI:** 10.1038/s41386-021-01147-7

**Published:** 2021-09-20

**Authors:** Andre Zamani, Robin Carhart-Harris, Kalina Christoff

**Affiliations:** 1 Department of Psychology, University of British Columbia, 2136 West Mall, Vancouver, BC Canada; 2 Centre for Psychedelic Research, Department of Brain Sciences, Imperial College London, London, UK

**Keywords:** Psychology, Consciousness, Cognitive neuroscience, Cognitive control, Human behaviour

## Abstract

The human prefrontal cortex is a structurally and functionally heterogenous brain region, including multiple subregions that have been linked to different large-scale brain networks. It contributes to a broad range of mental phenomena, from goal-directed thought and executive functions to mind-wandering and psychedelic experience. Here we review what is known about the functions of different prefrontal subregions and their affiliations with large-scale brain networks to examine how they may differentially contribute to the diversity of mental phenomena associated with prefrontal function. An important dimension that distinguishes across different kinds of conscious experience is the stability or variability of mental states across time. This dimension is a central feature of two recently introduced theoretical frameworks—the dynamic framework of thought (DFT) and the relaxed beliefs under psychedelics (REBUS) model—that treat neurocognitive dynamics as central to understanding and distinguishing between different mental phenomena. Here, we bring these two frameworks together to provide a synthesis of how prefrontal subregions may differentially contribute to the stability and variability of thought and conscious experience. We close by considering future directions for this work.

## Introduction

**Fig. 1:**
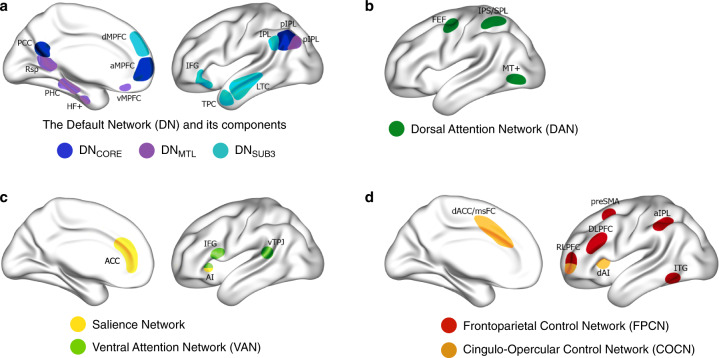
Large-scale brain networks with high relevance to the dynamic stability and variability of thought and conscious experience. Adapted from ref. [5]. **a** The default network (DN) is centered on the medial prefrontal cortex (mPFC), the posterior cingulate cortex and the lateral parietal cortex and extends into the temporal lobe and lateral PFC. Three subcomponents within the DN have been identified: (i) DN_CORE_ includes the anterior mPFC (amPFC), posterior cingulate cortex (PCC) and posterior inferior parietal lobule (pIPL), (ii) DN_MTL_ includes the hippocampal formation (HF), parahippocampal cortex (PHC) and a number of medial temporal lobe cortical projections, such as the retrosplenial cortex (Rsp), the ventral MPFC (vMPFC) and the pIPL, (iii) DN_SUB3_ extends more dorsally and includes the dorsomedial PFC (dMPFC), the lateral temporal cortex (LTC) extending into the temporopolar cortex (TPC) and parts of the inferior frontal gyrus (IFG). All three DN subcomponents seem to include subsections of the IPL. **b** The dorsal attention network (DAN) comprises a distributed set of regions centred around the intraparietal sulcus (IPS)–superior parietal lobule (SPL), the dorsal frontal cortex along the precentral sulcus near, or at, the frontal eye field (FEF) and the middle temporal motion complex (MT+). **c** The ventral attention network (VAN) comprises a ventral frontal cluster of regions, including the inferior frontal gyrus (IFG), the anterior insula (AI), and the adjacent frontal operculum (not shown); it includes the ventral temporoparietal junction (vTPJ). Although the VAN is predominantly right lateralized, a bilateral salience network (SN) has also been defined. The most prominent regions of the SN are the AI and the anterior cingulate cortex (ACC). **d** Two “control” networks have been discussed in the literature. The frontoparietal control network (FPCN) includes the dorsolateral PFC (DLPFC) and the anterior IPL (aIPL). Under a broader definition, the FPCN extends to regions including the rostrolateral PFC (RLPFC), the region anterior to the pre-supplementary motor area (preSMA), and the inferior temporal gyrus (ITG). The cingulo-opercular control network (COCN) includes the dorsal ACC (dACC)–medial superior frontal cortex (msFC) and bilateral AI–frontal operculum. The RLPFC contributes to both the FPCN and COCN. Not every region illustrated here is discussed in the present paper. Brain images were generated using BrainNet Viewer [354].

**Fig. 2:**
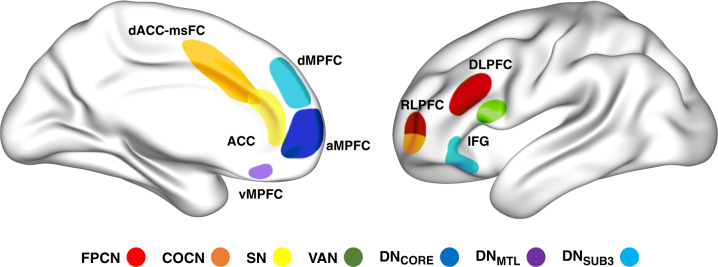
Prefrontal cortex subregions from major scale brain networks with high relevance to the dynamic stability and variability of thought and conscious experience. The dorsolateral PFC (DLPFC) is part of the frontoparietal control network (FPCN), while the rostrolateral PFC (RLPFC) is part of the FPCN and cingulo-opercular control network (COCN). The dorsal anterior cingulate cortex (dACC)/medial superior frontal cortex (msFC) is part of the COCN. The anterior cingulate cortex (ACC) is part of the salience network (SN). The inferior frontal gyrus (IFG) is part of the ventral attention network and third subcomponent of the default network (DN_SUB3_). The ventromedial PFC (vMPFC) is part of the medial temporal lobe subcomponent of the default network (DN_MTL_). The dorsomedial PFC (dMPFC) is part of the DN_SUB3_. The anterior medial PFC (aMPFC) is part of the core subcomponent of the Default Network (DN_CORE_). Brain images were generated using BrainNet Viewer [354].

The prefrontal cortex (PFC) is a broad swath of brain tissue encompassing numerous cytoarchitecturally and functionally heterogenous subregions. This heterogeneity has been explored throughout the last century of neuroscientific research, from cytoarchitectonic parcellations in the beginning of the last century [1], to functional connectivity-based large-scale brain network parcellations in the beginning of this century [2, 3]. Many large-scale brain networks (Fig. 1) include at least one prefrontal subregion in their canonical components (Fig. 2; see also Menon and D’Esposito, this issue [4]). Each of these networks is thought to influence cognition in relatively distinct ways [5], further underscoring the heterogeneity of the PFC. A wide range of mental phenomena are associated with the PFC, from goal-directed thought [6–11], to mind-wandering and spontaneous thought [5, 12–15], creative thought [16–19], rumination [20–22], and altered subjective experience under the effects of serotonergic psychedelics [23–31]. These various mental phenomena can be distinguished based on their neural, cognitive, and phenomenological correlates as we have recently argued in two theoretical frameworks: The dynamic framework of thought (DFT) [5] and the relaxed beliefs under psychedelics (REBUS) model [32].

The DFT and the REBUS model distinguish among the diverse PFC-related mental phenomena by highlighting a central dimension of thought and conscious experiences: its stability vs. variability over time. The DFT describes human thought as a sequence of dynamically changing mental states that range from being highly variable to being highly stable over time: highly stable thought dynamics feature a narrow range of contents separated by predictable transitions, whereas highly variable thought dynamics feature a diverse range of contents interspersed by relatively unpredictable transitions. The DFT also describes how alterations in large-scale brain network interactions may underlie changes in the variability or stability of thought. The REBUS model, on the other hand, suggests that serotonergic psychedelics induce a heightened variability of conscious experience (defined as pure phenomenological experience [33]) through their effect on brain-wide hierarchical information flow.

Put together, these two frameworks can provide a conceptual basis for understanding how different prefrontal subregions may contribute to increased stability or variability in thought and conscious experience. Here, we combine insights afforded by the frameworks in an attempt to do that. We begin by reviewing how the two frameworks theoretically approach the stability and variability of thought and conscious experience. We then present a summary of how the primary concepts put forth by either framework are related to the functions of individual prefrontal subregions and the large-scale networks they form. We close by integrating perspectives from the two frameworks and considering future research directions.

## Conceptual frameworks for understanding mental state dynamics

### Dynamic framework of thought (DFT)

The DFT is a conceptual framework for understanding human thought [5] that distinguishes between different kinds of thought by taking into account their dynamics of transition, in addition to their contents. The framework builds upon existing research on thought and mind-wandering that until recently primarily focused on the stimulus-independence and task-unrelatedness of mental state contents, to offer a more expanded account that brings into central focus the dynamics of mental states - or the manner in which those states change over time.

**Fig. 3:**
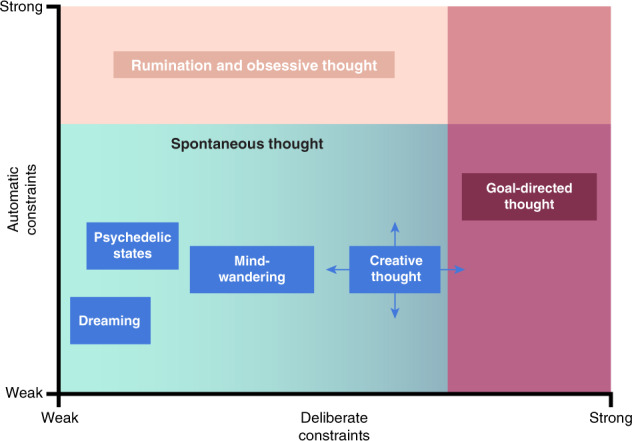
Visualization of the conceptual space for thought dynamics put forth in the dynamic framework of thought [5]. Deliberate and automatic constraints serve to limit the contents of thought and how these contents change over time. Deliberate constraints are implemented through cognitive control, whereas automatic constraints can be considered as a family of mechanisms that operate outside of cognitive control, including sensory or affective salience. Generally speaking, deliberate constraints are minimal during dreaming, tend to increase somewhat further during psychedelic states and more so during mind-wandering, increase further during creative thinking, and are strongest during goal-directed thought. There is a range of low-to-medium level automatic constraints that can occur during dreaming, psychedelic states, mind-wandering, and creative thinking, but thought ceases to be spontaneous at the strongest levels of automatic constraint, such as during rumination or obsessive thought. Creative thinking may feature pronounced oscillations between different modes of thought that feature varying amounts of automatic and deliberate constraints (indicated by arrows on the figure) [19].

The DFT proposes two general ways through which the dynamics of thought can be altered: deliberate and automatic constraints (Fig. 3). These constraints are distinguished based on their unique neural and phenomenological correlates and can increase the stability or variability of thought and conscious experience in different ways. A number of recent studies have used this dynamic view of thought to directly assess moment-to-moment changes in conscious experience through experience sampling [34–38].

Deliberate constraints are exemplified by cognitive control and executive processes that are supported by the brain’s control networks (Fig. 1; see also Menon and D’Esposito, this issue [4]). Such constraints primarily contribute to increased stability of thought over time by restricting thought contents and the transitions between them, typically in the service of an explicit goal [9, 11]. Consider a time when you may have misplaced your phone and were trying to remember where you last put it. You may have closed your eyes and began systematically recalling the order of places you visited before noticing the absence of your phone. In these circumstances, your thoughts would have been under high deliberate constraint, since their contents would have been restricted to being about places you visited that day, while transitions between them may have been predictable and logically constructive to completing the goal of finding your phone.

It is also possible, however, that deliberate constraints may contribute to an increased variability of thought in certain contexts, such as creative thinking. Creative thinking has recently been described as a dynamic process characterized by shifts between different modes of thought (as indicated by arrows in Fig. 3) and aimed at producing original and useful ideas [19, 39, 40]. Creative ability has been linked to executive capacity [41–43], and appears to reflect one’s ability for rejecting uncreative ideas [44–47], maintaining focus on internally generated thoughts against external distractors [45, 48], deliberately evaluating the quality of an idea [18], or implementing more creative but also more executively taxing idea generation strategies [30]. Each of these cognitive processes may contribute to increased variability, but only indirectly—by supporting the generation of a particular range of “more creative” thought contents. For instance, rejecting an uncreative idea could involve the deliberate pausing of the thought stream, so that when it resumes, a different range of potentially more creative ideas are generated. This would be an indirect contribution to increased variability since an intervening deliberate thought process redirected the overall direction of the creative process.

Automatic constraints, on the other hand, are a family of mechanisms that operate outside of cognitive control and have been tentatively linked to the brain’s salience networks and the core subcomponent of the default network (Fig. 1). Automatic constraints include mechanisms such as affective salience [49, 50], sensory salience [51], and habits [52], that can constrain thought variability. Automatic constraints primarily increase the stability of thought by making certain thought contents persist across multiple mental states. For example, you may recall a time when you were in conversation with a friend when thoughts about an unrelated, upsetting event that happened earlier in the day kept arising and grabbing your attention, making it difficult to focus on what your friend was saying. These thoughts were under high automatic constraint due to their affective salience and occurred without deliberate intention on your part (and possibly, despite your attempts to prevent them from arising). However, automatic constraints may also increase the variability of thought over time by supporting rapid switches of attentional focus between different stimuli when there is an abundance or a variety of salient information. For example, walking through a haunted house during Halloween is likely to involve multiple sudden loud noises that happen in quick succession, such that you quickly and repeatedly orient toward (or away) from their source.

The DFT holds that when both deliberate and automatic constraints are relatively low, thoughts arise and unfold more “spontaneously” and are marked by variability that is supported by the brain’s medial temporal lobe subcomponent of the default network (Fig. 1). During such “spontaneous” thought, there is increased variability of mental states due to diminished constraints on their contents and transitions. We recently argued [53] for a phenomenological and neurocognitive distinction between spontaneously generated thoughts and those that arise under automatic constraints: while both may feel spontaneous in the sense that the timing of their occurrence is unpredictable (since they are not deliberately generated), we proposed that thoughts will feel more spontaneous when their contents are also unpredictable. Automatically constrained thoughts may be experienced as *abrupt transitions* in the stream of thought—their arising may feel spontaneous due to the unpredictability of their timing of occurrence; however, their content is relatively predictable or at least easily explainable in relation to one’s current concerns, goals, motivations, and emotional states. More spontaneously generated thoughts, on the other hand, may be experienced as *wayward transitions* in the stream of thought; they may feel even more spontaneous, due to the unpredictability of their timing of occurrence as well as their contents, which may not bear obvious connection to latent cognitive variables or environmental context. Thus, spontaneously generated thoughts that arise through wayward transitions will likely generate a stronger subjective experience of spontaneity, since both the timing of their occurrence and contents are relatively unpredictable [53].

According to the DFT [5], when spontaneous thought dominates the thought stream, a wider range of mental states are likely to occur with less predictable transitions between them. An example of this may be found when one’s thoughts jump around from topic to topic during a boring lecture—thinking about a party that happened last week, to wondering what the outcome of an upcoming election will be, to thinking about what you will tell your parents when you see them later that day. These wandering thoughts feature distinct shifts in content and do not bear obvious connection with one another [53], thus forming a relatively spontaneous stream of thought that may result in increased variability of thoughts over time.

Dynamic interactions between different large scale brain networks are proposed to underlie the deliberate and automatic constraints, as well as the spontaneous thought processes identified in the DFT (Fig. 1). Below we elaborate on the original DFT framework, reviewing how specific prefrontal subregions from these networks may contribute to stability and variability in thought (Fig. 2). First, however, we provide an overview of the second conceptual framework that prominently focuses on the dynamics of conscious experience: the REBUS model [32].

### Relaxed beliefs under psychedelics (REBUS)

The REBUS model proposes a unifying account of how serotonergic psychedelic compounds affect conscious experience [32]. Serotonergic psychedelics are a specific class of psychoactive compounds including but not limited to lysergic acid diethylamide (LSD), psilocybin, and dimethyltryptamine (DMT), that principally act upon cortical serotonin 2A receptors [54–64] to achieve their profound psychological effects [32, 65, 66] and therapeutic potential [67–72]. Moving forward, we use the term psychedelics to refer to these serotonergic psychedelics.

**Fig. 4:**
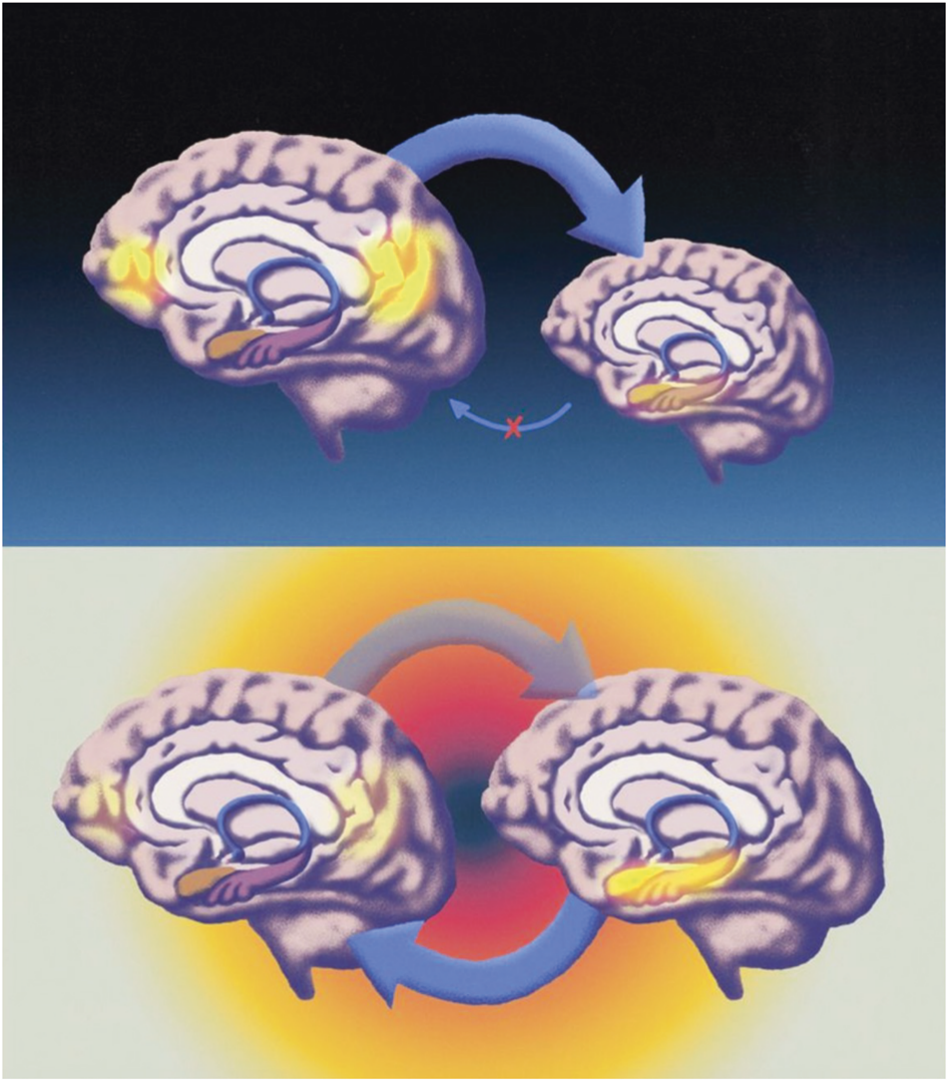
Schematic drawing of the brain mechanisms outlined in the REBUS model, adapted from ref. [32]. Top panel: brain organization in psychopathologies such as depression in which high-level beliefs or priors (e.g., instantiated by the Default Network Core) are overweighted (thick top-down arrow), causing a suppression of and insensitivity to bottom-up signaling (e.g., stemming from the limbic system). We show compromised bottom-up signaling via a thin arrow with a red cross over its center. Bottom panel: brain organization under psychedelics. The top-down arrow has been made translucent to reflect a deweighting or relaxation of high-level beliefs or prior (this component of the model is referred to by the acronym REBUS). The effect of this deweighting is to enable bottom-up information intrinsic to the system, to travel up the hierarchy with greater latitude and compass. That the two brains on the bottom panel are on the same level and of the same size is intended to reflect a generalized decrease in hierarchical constraints under the psychedelic. Illustrations by Pedro Oliveira, courtesy of Favo Studio.

According to the REBUS model, psychedelics contribute to an increased variability of conscious experience by significantly altering the brain’s neurocognitive hierarchies of information flow. Specifically, psychedelics are thought to relax the constraining influence that beliefs at higher levels of the brain’s neurocognitive hierarchies have on bottom-up information. This can also be described as a decrease in top-down and an increase in bottom-up information flow (Fig. 4), consistent with experimental findings of psychedelically-induced reductions to brain-wide top-down signaling [73], directed information flow [60, 74], and hierarchical information flow [29, 30]. Such alterations to hierarchical information flow may play a role in creating the increased variability or diversity of brain activity that is observed during psychedelic states [75–80], given that bottom-up information flow is no longer as constrained by top-down sources. In other words, a diversity of information that is usually compressed out of conscious awareness is now available to register more fully within it.

The term “belief” as used in REBUS does not refer to the limited range of conscious propositional stances held toward the world. Instead, REBUS uses “belief” more broadly, as a synonym for the more technical Bayesian term ‘prior’, referring to predictions encoded in neuronal connections and activity. An alternative term would be ‘assumption’. REBUS directly borrows these concepts from a hierarchical predictive coding view of brain function [81–84].

Hierarchical predictive coding casts the brain as a predictive system whose numerous hierarchical levels operate to predict the input they are about to receive from their respective lower levels. Incoming information can originate exteroceptively [82, 85, 86], interoceptively [87, 88], and in the stream of thought [53, 89–91]. These predictions serve to “compress” the incoming information and allow the brain to avoid redundancy by processing only the unpredicted portions of information, known as prediction error. The confidence in predictions can be high or low and can be continuously adjusted based upon the expected reliability and relevance of incoming information—a process called “precision-weighting”, or the flexible assigning of weights to predictions relative to prediction errors. A related formulation, the free-energy principle, casts the brain as always striving to decrease the difference between its predictions and incoming information -- in other words, minimizing prediction error [82, 92]. To minimize prediction error, the brain can either modify its predictions or act to adjust incoming information so that it may better fit its predictions [82, 83, 92]. A more in-depth discussion of hierarchical predictive coding can be found elsewhere [32, 84, 92, 93].

Another important conceptual component of the REBUS model is the entropic brain hypothesis [94, 95]. According to the entropic brain hypothesis, there is a close mapping between the diversity or richness of subjective conscious experience and the entropy of spontaneous brain activity. Entropy (in an information theoretic sense) is a measure of the unpredictability of information over time, with greater uncertainty equalling greater entropy. This mapping allows conscious states to be differentiated based upon the degree of entropy in their underlying brain activity. For instance, highly entropic brain states are hypothesised to reflect informationally rich experiential states. Psychedelic states are one such kind, featuring an increased diversity and flexibility of subjective experience [65, 96] as well as highly entropic underlying neural activity [75–80]. In contrast, other conscious states feature a relatively diminished richness and flexibility of subjective experience, along with highly predictable brain dynamics (i.e., low entropy). These include clinically relevant states such as rumination and obsessive thought that have been associated with lower than typical spontaneous brain entropy across time [97] and a reduced richness of associated conscious experience [5, 20]. However, the quintessential low entropy states are states of unconsciousness that fall outside of the “critical zone” for conscious experience [95] (the zone within which consciousness arises, and within which brain activity is neither too ordered nor too disordered in terms of its degree of entropy).

In summary, the REBUS model proposes that psychedelics increase bottom-up information flow by decreasing the compressing influence of implicit top-down beliefs (i.e., reducing the precision-weighting for predictions), leading to an overall increased variability of conscious experience. At the neural level this corresponds to decreased top-down signaling and increased informational entropy of underlying neural activity. The REBUS model reflects a growing body of functional neuroimaging findings that show the effects of psychedelics on major scale brain networks and their prefrontal nodes [23–25, 98–100].

### Synergies between the frameworks

The DFT and REBUS model exhibit a variety of conceptual synergies. First, both frameworks treat cognitive control as a process that stabilizes thought and conscious experience. Within the DFT it is described as deliberate constraint upon thought, while the REBUS model describes it as deliberate influence on the psychedelic state that can be disruptive to positive therapeutic changes (e.g., when the experiencer deliberately strives to suppress their altered state). Indeed, the standard protocol for psychedelic therapy encourages participants to “let go” or “surrender” to the experience [101, 102], which could be interpreted as suspending deliberate constraints upon cognition. Both frameworks also posit that there are processes outside of cognitive control that contribute to increased stability of thought through some form of automatic influence. These include but are not limited to affective salience, habits, and various neurocognitive aspects of the sense of self [5, 19, 32]. In the DFT such processes are termed automatic constraints, whereas the REBUS model describes them as implicit top-down beliefs.

The presence of increased constraints or implicit beliefs, however, does not necessarily result in decreased variability of thought and conscious experience. For example, a recent update of the DFT describes how some automatic constraints, such as affective salience, may contribute to *increased* variability of thought and conscious experience [19]. Affective salience refers to the emotional, conceptual, and personal significance elicited by a stimulus (perceptual or otherwise), that could trigger non-volitional attention toward it [5]. Similarly, the REBUS model proposes that the precision-weighting (i.e., confidence) of high-level constraining beliefs is decreased under psychedelics, which can enable ordinarily compressed mental contents to register more readily in conscious awareness, in turn making the conscious experience more variable and less stable [32]. Consistent with this, psychedelic states have been shown to feature increased emotionality lability and release [65, 103–106]. This quality may contribute to increased variability due to emotional processing being freed from higher-level beliefs and becoming more easily influenced by a variety of endogenous or exogenous stimuli (e.g., ascending affect or bodily sensations, thoughts, or sensory perceptions). In other words, implicit beliefs can still constrain experience under psychedelics (such as those related to affect) but may instead contribute to an increased variability of thought and conscious experience in doing so.

## How do different prefrontal subregions contribute to stability and variability?

Here we organize the evidence in response to this question around the central concepts from DFT and REBUS: deliberate constraints, automatic constraints, and top-down beliefs. We describe how different prefrontal subregions may contribute to increased stability or variability in thought and conscious experience in relation to these concepts. In particular, we focus on prefrontal subregions that are part of the frontoparietal control network, cingulo-opercular control network, salience network, ventral attention network, core subcomponent of the default network, medial temporal lobe subcomponent of the default network, and the third (or dorsomedial PFC) subcomponent of the default network (Figs. 1 and 2).

Even though here we focus on specific prefrontal subregions, each large-scale brain networks is affiliated with multiple regions and brain structures inside as well as outside of the PFC that also make crucial contributions to stability and variability of thought (Fig. 1) [5, 32]. Moreover, we hold that the functions of any one brain region only emerge in relation to its interactions with other areas of the brain. This perspective has become prevalent in the field of cognitive neuroscience over the past decade, with work increasingly focusing on major scale brain networks [2, 3], brain activity configuration states [99, 107, 108], and whole-brain information dynamics [75–78, 97]. However, we also hold that it is important to recognize how individual regions play relatively distinct and important roles in cognition. We invite our readers to maintain this balance in perspectives as they read on.

### Deliberate constraints

Deliberate constraints are closely linked to recruitment of the brain’s frontoparietal and cingulo-opercular control networks (Fig. 1). These networks support the flexible, top-down implementation of cognitive control and executive function [5, 11, 109–111] through dynamically coupling with other brain networks. For example, to implement cognitive control over external versus internally generation information, the frontoparietal control networks can couple flexibly with the dorsal attention network and the core subcomponent of the default network, respectively [11, 112–115]. The two control networks may also contribute to cognitive control at different timescales: the frontoparietal control network may be preferentially engaged for shorter-term processes such as adjusting and initiating cognitive control [116, 117], while the cingulo-opercular control network seems to support longer-term processes such as maintaining task sets over time [109] and monitoring performance [118].

Deliberate constraints may primarily contribute to an increased stability of thought by constraining the contents and transitions between thoughts in line with current goals or task demands. This is associated with the recruitment of multiple prefrontal control network regions, including the dorsolateral PFC (DLPFC), rostrolateral PFC (RLPFC), and dorsal anterior cingulate cortex (dACC; Fig. 2). The DLPFC is considered to be a part of the frontoparietal control network, the dACC is part of the cingulo-opercular control network, and the RLPFC participates in both control networks [5].

#### Frontoparietal control network

One way in which the frontoparietal control network may contribute to stronger deliberate constraints is through the maintenance and implementation of rules, a process that has been associated with DLPFC or RLPFC recruitment. Rules refer to conditional associations between stimuli and actions that should be selected or suppressed as a function of context [119] (e.g., if the pedestrian signal is on, then walk across the crosswalk; if the pedestrian signal is off then do not walk across). Rules may increase stability in thought and conscious experience by biasing thought’s contents toward a particular range of behaviors and cognitive process. At the neural level, this is thought to be implemented through the PFC’s biasing influences on the neural activity of other regions throughout the brain [120] (see also Freidman and Robbins, this issue [121]).

Some rules, however, such as those pertaining to long-term personal goals, are linked to memory contents rather than external environmental contextual cues, and thus depend upon memory retrieval for their implementation. The DLPFC appears to support this through the online maintenance of the relationship between rules and the expected value of outcomes, as derived from memory. The RLPFC, on the other hand, may represent multiple rule-outcome associations and their higher order integrations [119] to help guide adjustments in goal-directed cognition [119, 122, 123]. This functional distinction between DLPFC and RLPFC is consistent with a rostrocaudal gradient of PFC organization marked by increasing levels of abstraction in processing moving anteriorly from the DLPFC to the RLPFC [123–126].

Consistent with this, increased regional cerebral blood flow to the DLPFC is associated with expecting cue onset during rule-based behavior [127], while focal damage and inhibitory transcranial magnetic stimulation to the DLPFC are linked to deficits in “prospective memory” ability [128, 129], or the ability to realize delayed intentions [130]. The DLPFC thus appears to support rules that apply beyond the immediate moment, so that cognition and behavior may adhere to them across changing contexts. This may also extend to circumstances in which the external environment contains cues that are counterproductive to desired long-term outcomes. For example, increased activation of the DLPFC is associated with effortfully resisting a smaller shorter-term erotic reward over greater longer-term rewards [131], while disruption to the DLPFC through inhibitory transcranial magnetic stimulation impairs the prioritization of greater long-term financial rewards over lesser short-term ones in temporal discounting tasks [132] and increases risky decision-making during gambling [133]. These findings suggest that the DLPFC may underlie the deliberate shielding of long-term goals from possible distractors, potentially by retrieving valued, goal-congruent rules from memory.

The RLPFC, on the other hand, appears to act as a functional intermediary between the frontoparietal and cingulo-opercular control networks [134], representing multiple rule-outcome associations at once that may help to guide adjustments to cognitive control [119]. This has been referred to as “cognitive branching”—the ability to put alternate courses of action on hold [135]. RLPFC activation has been associated with anticipating and planning for future failures of rule-adherence [131] and maintaining task subgoals [136], suggesting that the RLPFC is not as involved with directly implementing rules as it is with maintaining multiple options. RLPFC activation has also been found to increase over the course of carrying out a sequence of tasks and to peak during the final task of this sequence, while transcranial magnetic stimulation to the region increasingly disrupts task performance the further along participants are in the sequence [137]. These results suggest that the RLPFC influences task performance the most when task knowledge is less strongly encoded, such as during the later parts of a task sequence, perhaps through its representation of multiple possible rules.

Altogether, the DLPFC may increase deliberate constraints upon thought by implementing memory-derived rules based upon the expected value of their outcomes (with goal completion being highly-valued), while the RLPFC represents multiple rule-outcome relationships simultaneously to help guide rule selection. These rules may contribute to increased stability in thought and conscious experience by specifying a particular range of behaviors and cognitive process to occur over others.

Thought suppression may be one example of this. During thought suppression unwanted thoughts are purged from one’s conscious experience (e.g., Anderson and Floresco, this issue [138]). Certain thoughts may hinder progress toward long-term goals and limiting their occurrence through mechanisms such as thought suppression may benefit achieving those long-term goals. For example, one study [44] had participants were instructed to complete a verb generation task that involved generating novel verb associates in response to visually presented nouns. Participants had already seen some of the nouns previously as part of uncreative (or commonly associated) noun-verb pairs that they were told to study for a later memory recall test. This manipulation was intended to create low- and high-constraint nouns, with the latter already associated with common verbs. When generating novel verbs to highly constrained nouns, participants exhibited increased functional connectivity between the DLPFC and default network core, which the authors interpreted as evidence of increased top-down inhibition to reject (or suppress) the pre-potent uncreative verbs.

Other studies also show the DLPFC is closely associated with thought suppression [139], exhibiting increased activation alongside decreased hippocampal activation during rule-based thought suppression [140–142]—effects that are greater when a thought is actively purged from conscious awareness [143]. Results from dynamic causal modeling and structural imaging indicate that top-down directed connectivity from the DLPFC to the hippocampus best captures the DLPFC’s role in thought suppression [139, 143, 144] (see also Anderson and Floresco, this issue [138]). Hippocampal processes have been linked to increased variability in thought [5, 145] and the generation of spontaneous thought contents [15, 146]. Therefore, the DLPFC may influence hippocampal generative processes by constraining them to bias the stream of thought toward contents in line with current goals and task demands [5]. An interesting implication of this view is that thought processes themselves may be understood as internal behaviors that can be governed by rules.

The RLPFC may also affect thought dynamics, through its representation of multiple rule-outcome associations. Another study [147] that employed a novel verb generation task only manipulated whether participants were instructed to “think creatively”. Verbs generated following this simple manipulation were measured to be more creative using latent semantic analysis (a method for measuring the creativity of an idea based upon its semantic relationships to other concepts) and were associated with increased right RLPFC activation and increased functional connectivity between the RLPFC and the anterior medial PFC node of the default network core. This result is distinct from the increased functional connectivity between the DLPFC and default network core proposed to be associated with thought suppression, following the above described noun-verb pairing manipulation [44].

This difference in DLPFC vs. RLPFC connectivity with the default network core between these manipulations is consistent with the previously described difference in function between DLPFC and RLPFC according to level of abstraction in cognitive control: instructions to “think creatively” are more abstract and supraordinate than the rules that may have guided the rejection of primed, highly common verbs, and specific ideas. Such supraordinate-level information about how to “think creatively” may have included multiple rule-outcome associations connecting different thought dynamics to valued outcomes, perhaps including different executively demanding idea generation strategies that may be alternated [43]. Their implementation would increase stability in thought and conscious experience by organizing sequences of thought around current goals, and be associated with increased RLPFC recruitment.

It is possible, however, that deliberate constraints supported by the frontoparietal control network may also contribute to increased variability in thought during creative thinking. For example, the frontoparietal control network has been found to “drive” brain dynamics toward unique network configurations that favor the generation of highly creative ideas [148]. This may correspond to the deliberate initiation of generative modes of thought that feature more variable and novel thought contents [39], corresponding to an increased variability of thought and conscious experiences [19]. Even in these contexts though, the control networks may play a stabilizing role by increasing deliberate constraints upon thought and restricting the range of thought contents and dynamics (i.e., focusing thoughts to be about a particular topic or unfold through a certain idea generation strategy). Thus, the control networks may primarily contribute to an increased stability in thought and conscious experience at more global timescales, although local short-term increases in variability (e.g., when new ideas or possible moves in a problem solving task are briefly entertained for their suitability) may also be implemented.

#### Cingulo-opercular control network

The cingulo-opercular control network supports deliberate constraints in ways that may be distinct yet complimentary to that of the frontoparietal control network. While the frontoparietal control network primarily underlies deliberate constraints that seem to operate on shorter timescales, the cingulo-opercular control network appears to support deliberate constraints at relatively longer timescales. This is suggested by the functions associated with RLPFC and dorsal ACC (dACC; Fig. 2) [5].

As discussed in the previous section, the RLPFC is considered a member of both control networks [5] and appears to act as a functional intermediary between them [134]. This includes representing multiple rule-outcome or action-outcome relationships simultaneously [119] that can help guide adjustments of cognitive control on short [122] as well as long [137] timescales. It may contribute to increased stability in thought and conscious experience by scaffolding mental contents through abstract, super-ordinate goal representations [123–125].

Deliberate constraints are also thought to be supported by the dACC (extending into the medial superior frontal cortex; Fig. 2), a region considered part of the cingulo-opercular control network [5, 117]. An early influential model suggested that the dACC is preferentially involved in performance monitoring, response conflict, and error detection for the purpose of adjusting cognitive control [149–152]. This early model, however, did not account for subsequent observations of robust dACC activation across situations that do not involve explicit control demands, including pain, threats, and rewards [153–158]. A more comprehensive account of dACC function has subsequently emerged, suggesting that dACC recruitment underlies the valuation of actions and online adjustment of behavior to meet current contextual demands [156, 159–161] in service of goal attainment [162] (see also Monosov and Rushworth, this issue [163]). This proposed function appears enabled by the dACC’s rich interactions with control, motor, and visceral neural systems.

Anatomical connectivity between the dACC and frontoparietal control network [164] likely provides the dACC with access to goal-related information. Meanwhile, anatomical connectivity to numerous cortical motor regions as well as the spinal cord [165–168] may enable the dACC to modulate outward behavior and update the expected outcomes of actions. Neural activation in the dACC exhibits a somatotopic organization, with actions related to different motor effectors (i.e., tongue, hand) recruiting distinct portions of the dACC during reward contingency learning [169]. Meanwhile, dACC neural activation is also modulated by the trade-off between an action’s expected reward and its expected effort costs [170], underscoring the role of dACC in linking actions to expected outcomes.

Lastly, anatomical and functional connectivity to the anterior insula [3, 171–175] may allow the dACC to interact with and exert a biasing influences upon viscero-somatic processing to prepare the body for actions [169]. When processing interoceptive information from the viscera (e.g., a noxious tactile stimulus), dACC recruitment is modulated by one’s belief in stimulus controllability [176]. This may imply the dACC processes interoceptive information relative to control-related beliefs about how much effort is needed to achieve a goal (e.g., remove pain). Indeed, individuals with anorexia nervosa, an eating disorder characterized by an excessive fixation on bodily thinness [177], exhibit increased resting state functional connectivity between the dACC and the retrosplenial cortex of the medial temporal lobe subcomponent of the default network [178]. This may reflect a heightened self-relevance of goals related to one’s body [162] and may account for the increased tendency of individuals with anorexia nervosa to engage in outcome-oriented imagination about their body, given that the medial temporal lobe subcomponent of the default network is associated with the generation of perceptually detailed thought contents [179].

In summary, the dACC may contribute to increased deliberate constraints by aligning outward actions, along with those made toward the internal stream of thought (e.g., in the case of outcome-oriented thoughts in anorexia), with one’s goals. This may contribute to an increased stability of conscious experience in contexts when the value of one action clearly outweighs the value of other possible actions, and when this difference in values remain relatively stable over time. Indeed, the dACC has been argued to underlie “tenacity”—the ability to maintain a consistent pattern of behavior over time [162]. In individuals with anorexia nervosa for instance, the expected value of being slim may be so heightened [177] that patterns of behavior aimed toward the goal of maintaining a low body weight occur with very high frequency. However, in an uncertain or rapidly changing environment, when multiple actions have interchangeable value or an action’s value changes drastically over time (e.g., from having a highly positive to highly negative reward value), the constraints exerted by the dACC may contribute to reducing the stability of thought and conscious experience and increasing its variability over time. This may occur, for example, as multiple actions are being simultaneously contemplated and the organism is in a state of indecision as to which action would be worthwhile to engage in.

### Automatic constraints

Automatic constraints are associated with the functions of multiple brain networks, including the default network with its three subcomponents, the salience network, and the ventral attentional network [5]. Like deliberate constraints, automatic constraints may primarily contribute to increased stability in thought and conscious experience although they may also lead to increased variability in certain contexts. Different prefrontal regions from these networks contribute to this in unique ways.

#### Default network core

Around the turn of the 21st century, a set of brain regions became identified for being consistently deactivated during external task demands but being consistently activated when such external task demands were reduced [180]. These regions described to underlie a “default mode” of human brain function [181] and eventually named the “default mode network” [182].

Scientific understanding of the default network has evolved considerably in the last two decades. It is no longer thought of as a “task-negative network”, but is instead understood to support a variety of internal mental processes [5, 20], including mind-wandering [14], autobiographical planning [11, 113], creative thinking [16, 18, 183–185], dreaming [186], and rumination [187–189], as well as aspects of self-experience [23, 24]. Indeed, default network activation is task-positive when the experimental task requires internal-oriented mental processes, including when these processes are goal-directed [11]. The default network is also now thought to be comprised of three distinct subcomponents (Fig. 1): a core subcomponent, medial temporal lobe subcomponent, and third (or dorsomedial) subcomponent [5, 190]. The core subcomponent appears recruited during the widest variety of experimental paradigms [20].

The core subcomponent of the default network (DN_CORE_) is comprised of the anterior medial PFC, posterior cingulate cortex, and inferior parietal lobule, each of which are highly interconnected with many other brain regions [5, 90, 190]. It serves as a site of integration between the other two subcomponents [190] because of which some have even argued that it may not be a true network [191]. It is associated with a diverse range of functions, including the mnemonic elaboration of thought contents [15, 18, 20, 192], the coordination of highly abstract and large receptive fields of the world known as event models [90, 192], and self-referential thinking [193–198].

The anterior medial PFC (aMPFC) is the anterior-most node of the DN_CORE_ (Figs. 1 and 2). It acts as a site of integration for prefrontal nodes from the other default network subcomponents [191] and is closely associated with abstract self-referential processes [170, 193–195]. This has led to proposals that DN_CORE_ recruitment underlies increased automatic constraints on thought [5] that bias the stream of thought toward personally significant information [20].

Consistent with this, activation in aMPFC increases as a function of the self-relatedness of ongoing processing. This includes increased activation when judging whether an adjective applies to oneself [193], which also increases as a function of the perceived self-relevance of a considered personality trait [194]. Such activation is diminished when considering whether an adjective applies to an intimate other [195] and further diminished for a non-close other [196]. Neural activation in the aMPFC is also greater when considering how personality traits may apply to oneself in the present than it is when judging how they did in the past or may in the future [197].

These findings all involve the manipulation of relatively abstract self-related information: adjectives and personality traits are broad descriptors that generalize across specific information about a person [199]. This may imply that the aMPFC preferentially supports self-referential processing information that is more abstract. Indeed, when participants evaluate whether listed traits apply to themselves, aMPFC activation has been found to increase more for context-invariant traits (i.e., “In general, I am…”) than context-dependent traits (i.e., “At school, I am…”) [198]. The aMPFC thus appears to underlie self-processing at a more schematic level.

Schemas refer to complex and abstract knowledge structures formed by extracting and generalizing statistical regularities across previous individual experiences [200, 201]. They enable present-moment experience to be scaffolded in relation to generalized knowledge [192, 202], suggesting a potential role for schemas in increased stability of thought and conscious experience. In general, the medial PFC is thought to be involved in schematic information processing more so than posterior brain regions [90, 192]. Of the medial PFC’s multiple subregions, the ventromedial PFC appears to be the most crucial for schema formation and implementation [203], while the aMPFC may support self-referential schema more specifically. Therefore, the aMPFC may contribute to an increased stability of thought and conscious experience by automatically constraining thought contents using abstract self-referential schemas toward personally significant information.

For example, an excessive biasing towards abstract, personally significant thought contents may be a characteristic feature of ruminative thought [204–208]. Rumination refers to a style of thinking that is highly common in major depressive disorder [206]. It includes repetitive [209], over-general (or schematic) [210–212], and personally significant thought contents often about the perceived causes of one’s depression [204–208], that arise with relative automaticity [5, 20]. It is considered to be a highly constrained, rigid, and stable type of cognition featuring a limited range of mental states [5, 20, 94, 95]. It is also closely linked to aMPFC function [5, 20, 188, 213, 214]. Increased functional connectivity between the aMPFC and DN_MTL_ has been observed during induced rumination in healthy controls [188], while less variable patterns of functional connectivity between the aMPFC and DN_MTL_ were found in depressed individuals during a resting-state [213]. These findings suggest that during self-focused rumination, the aMPFC may increase automatic constraints on thought by modulating the mnemonic contents and dynamics generated by the DN_MTL_ to fit with negative self-schemas. Such negative self-schemas may engender stereotypic sequences of thought, marked by highly predictable contents and manners of unfolding.

#### Default network medial temporal lobe

The medial temporal lobe subcomponent of the default network (DN_MTL_) spans the hippocampus, parahippocampus, retrosplenial cortex, posterior inferior parietal lobule, and ventromedial PFC (Fig. 1) [5]. It is associated with episodic thinking [183, 215–217], the generation of specific, contextual, and visuo-spatially detailed thought contents [20, 179, 190, 192], as well as the generation of spontaneous thoughts: neural activity, especially in its hippocampal components, has been found to precede Vipassana meditators’ reports of a thought arising during meditative practice [15] and the open recall of previously viewed video clips [146]. The DN_MTL_, through its hippocampal, parahippocampal, and retrosplenial components, is thought to contribute to increased variability in thought and conscious experience by (re)activating hippocampal-neocortical neural ensembles and enabling mental state transitions through associative cueing between mental contents [5, 145]. This is especially pronounced when deliberate and automatic constraints are low [5].

In contrast to the medial temporal lobe structures located in the DN_MTL_, the ventromedial PFC (vMPFC) may support *increased* automatic constraints on thought. Since the label “vMPFC” is somewhat inconsistently used in the literature [170, 201], here we employ the label “medial orbitofrontal cortex (mOFC)” in its place, referring to the specific region encompassing Brodmann area 14 and the medial portion of Brodmann area 11 [170].

The mOFC appears to underlie emotional evaluations about the value of internally generated events, such as spontaneous thoughts, based upon their relevance to current goals and needs [170]. This view is supported by multiple lines of evidence. First, the mOFC exhibits strong anatomical connections [218, 219] and functional connectivity [190, 220] with memory-related regions in the DN_MTL_ such as the hippocampus, parahippocampus, and retrosplenial cortex (see also Haber et al., this issue [221]), that support the construction of mnemonic thought contents [15, 190, 222–225]. Second, the mOFC is anatomically connected to brain regions that likely provide it with access to information about different goals and needs, including: lateral prefrontal regions [174, 175, 226, 227] for task context and long-term goals [119, 122], the anterior medial PFC [227, 228] for self-relevance and personal significance [193–195], and various subcortical nuclei [227–231] for information about reward, punishment, and interoceptive signals concerning physiological demands [232–234]. And lastly, neural activation in the mOFC increases during a variety of internally-oriented cognitions [14, 190, 191, 235–239] and correlates with the intensity of affective appraisals made toward thoughts [224, 236, 240].

Activation in mOFC positively correlates with the degree of rated familiarity and anticipated pleasantness for future episodic simulations [236], rated pleasantness for recalled episodic memories [224], and reported motivation to engage in autobiographical reflection [240]. This may imply that the mOFC supports the elaboration of thoughts in value-dependent ways, so that thoughts associated with higher estimated value may be more likely to be sustained over time [170]. For example, mOFC activation predicts individual differences in optimism bias while updating one’s explicit beliefs in response to good news over bad news [241]. This suggests that the mOFC may bias explicit belief construction and fixation based upon the value of self-confirmation. Meanwhile, findings from magnetoencephalography show how neural activity in the mOFC influences that of the anterior hippocampus during the beginning of imaginative scene construction [239]. This suggests that in certain contexts the mOFC may cue or bias the generation of mnemonic contents by the hippocampus. Thus, the mOFC may contribute to increased stability in thought and conscious experience by enacting automatic constraints that support the elaboration of highly valued sequences of thought, perhaps over other lesser-valued ones.

The mOFC’s role in automatic constraints may also be framed in terms of its association with schema-guided memory processes. The mOFC, as well as vMPFC more broadly, is closely linked to schema construction and maintenance [203]. For instance, lesions to the mOFC preserve the capacity for autobiographical memories to be cued but disrupt the ability to elaborate cued memories into extended sequences of thought. This is observed during past- and future-oriented autobiographical thinking, especially during fictitious thinking [242–245]. Fictitious thinking includes sequences of thought content that have not been episodically experienced, making it heavily reliant upon schemas to fill and guide its content. It may also rely upon schema for its initiation since it cannot be episodically cued. This explains why fictious thinking is more disrupted by lesions to the mOFC than other types of thinking that may have stronger episodic basis.

Thought that is heavily schema-dependent may rely upon interactions between the mOFC and the hippocampus during initial construction, suggesting that the mOFC may serve to provide an inceptive schematic-template to help guide thought generation [239]. Such a template may also help bolster hippocampal encoding for uncertain memories: increased functional connectivity between the mOFC and hippocampus has been observed after the viewing of movie conclusions that were preceded by temporally scrambled movie intros, compared to unscrambled ones [246]. This may imply that when the details of a new memory are uncertain (such as its temporal structure), schemas help to stabilize the initial encoding of the memory by filling in any gaps.

Combining its affiliations with value-based and schema-dependent thought, we suggest that the mOFC may contribute to an increased stability in thought and conscious experience by automatically constraining cognition and behavior around highly valued schemas. For example, if someone is planning to propose marriage to their significant other, they may be prone to engage in future-oriented thought about the proposal when even the slightest cue appears given the high value of the topic. This line of thought may be heavily dependent upon schemas for its elaboration since it is future-oriented and is likely to be structured by predictable cultural narratives (or schemas) about how the proposal ought to occur. As such, mOFC recruitment may underlie increased automatic constraints on thought by making highly valued thoughts more likely to be elaborated upon while also organizing their associated streams of thought around formularized schema. This is likely to result in a concomitant increase of stability in thought and conscious experience.

#### Default network third subcomponent

The third subcomponent of the default network (DN_SUB3_), also known as the dorsomedial subcomponent, spans the temporopolar cortex, lateral temporal cortex, posterior inferior parietal lobule, inferior frontal gyrus, and dorsomedial PFC (Fig. 1). It is the least well-understood of the default network subcomponents [5, 20], and appears to be implicated in conceptual processing [215, 247–249] and mentalizing [5, 170, 215, 216, 250, 251], or thinking about the mental states of others [252]. While the DN_SUB3_ is often characterized in terms of its sociocognitive functions, research has begun suggesting that the network may be better understood in terms of its involvement in constructive mental simulation in general, of which abstract social cognition is just one [216, 247, 249, 253].

This is also the case for the dorsomedial PFC (dMPFC) node of the DN_SUB3_ (Fig. 2), which is most often discussed in terms of its recruitment during abstract sociocognitive processes. This includes visual perspective taking [254, 255], evaluating why versus how someone performed a behavior [256, 257], differentiating in-group versus out-group individuals [258], maintaining knowledge of psychological traits [170, 259, 260] and social stereotypes [261], and forming impressions of others [262, 263]. However, the dMPFC is also linked to abstract non-social processes: It exhibits greater activation during the formation of high-construal relative to low-construal categories for both social and non-social stimuli [247, 249]. It is also activated when reading text passages that are abstract, social, or a conjunction of the two [248]. Finally, it is activated during abstract self-referential processes, showing greater activation when reflecting upon the meaning of autobiographical memories than when remembering them directly [240].

Similar to its parent network (the DN_SUB3_), the dMPFC thus appears to underlie abstract, highly constructive processing more broadly, such that its close association with social processing may only reflect the high frequency with which social information is encoded and processed at an abstract level [216, 247, 249, 253]. For instance, the dMPFC has been argued to underlie the abstraction of meaning from memories [264], and especially their emotional meaning [265]. The emotional meaning of a memory may be understood as its emotional gist, where gist refers to the coarse-grained global features of a *single* event, in contrast to the coarse-grained global features of *multiple* episodes referred to by the term schema [203]. A gist disregards the low-level features of an event so that the relationships between multiple diverse stimuli may be made more salient [249], allowing behavior and cognition to become organized around higher-level properties of the world. In terms of emotional gist, increased neural activation in the dMPFC is associated with greater vividness of recollection when recalling negatively-valenced episodic memories in younger adults but positively-valenced episodic memories in older adults [266]. This may imply that the dMPFC helps to coordinate the recollection of a memory through its associated emotional gist, depending upon an observer’s motivations or goals at the time of retrieval. As age increases, motivational priorities tend to shift so that negatively-valenced emotional experiences are downregulated in favor of positively-valenced ones [267], potentially accounting for the age-dependent differences in valenced memory-retrieval associated with dMPFC activation [266].

Altogether, this suggests that dMPFC recruitment increases automatic constraints on thought by augmenting the extent to which abstracted meaning, or gist, is used to guide the internal stream of thought. In the case of emotional memory, for example, this may contribute to increased stability in thought and conscious experience due to an affective gist “binding together” various mnemonic contents into a more stable recollected sequence that is biased by a particular affect.  In a similar way, it is possible that the dMPFC’s close association with social processing may reflect how when simulating another person in imagination, the internal stream of thought is constrained by an abstracted gist of a person.

Indeed, neural activity underlying the imagination of a famous person, especially in the dMPFC, has been found to be best reconstructed by a model that summates the neural activity underlying the imagination of different possible mental states depending upon how much a famous person is believed to occupy those states as rated by online survey takers [268]. These findings suggest that one way the brain may simulate and differentiate people is in terms of abstract mental state dimensions—akin to the “gist of a person”—that bind their lower-level features, such as common behaviors, into a stable representation [199, 269]. This may contribute to increased stability in thought and conscious experience by ensuring that different people are mentally simulated in automatically constrained and predictable ways.

In other instances of social cognition, however, the automatic constraints afforded by the dMPFC may contribute to increased variability in thought and conscious experience, such as in schizophrenia. Schizophrenia is a clinical condition associated with altered dMPFC neural activity [270–274], morphology [273, 275], and metabolism [276], that has been argued to feature a significantly increased variability of thought and conscious experience: common positive symptoms include hyper-associative thinking, auditory hallucination, and an attenuated self-other boundary [94, 95]. An attenuated self-other boundary refers to a diminished distinction between information related to oneself and others—a phenomenon that is specifically linked to altered activation of the dMPFC [271].

Hyperactivation of the dMPFC is associated with schizophrenic individuals’ propensity to endorse social values they had previously reported not to hold, following their exposure to individuals who acted as if they did hold them [271]. This suggests that for schizophrenic individuals, hyperactivity in the dMPFC may underlie an attenuated self-other boundary by introducing abstract information about social others (such as their values) into judgements about oneself. Indeed, compared to healthy controls, schizophrenic individuals have been found to commit more altercentric errors during a perspective taking task (i.e., accidentally and automatically computing another person’s visual perspective when making explicit judgements about one’s own) [277]. Furthermore, severity of positive symptoms is associated with improved visual perspective taking when one’s own perspective is consistent with that of another’s but worse performance when perspectives are inconsistent. This suggests that schizophrenic individuals may process self and other perspectives concurrently, and that this may facilitate or impair behavior depending upon the context [277]. It is possible that a similar process may occur when schizophrenic individuals misattribute their own behaviors or thoughts to others.

Thus, the dMPFC may contribute to an increased variability of thought and conscious experience in schizophrenia by confounding abstract self- and other-related information in judgements. Indeed, compared with healthy controls, schizophrenic individuals display less difference in the amplitude of anterior medial PFC neural activation, a brain region closely linked to self-referential processing [193–195, 198] and automatic constraints [5, 20], when listening to verbalized text passages recorded by themselves versus another person [278]. Schizophrenic individuals, therefore, may not segregate high-construal self- and other-information to the same degree as people without schizophrenia, which may disrupt the stabilizing effect that a normative sense of self has over cognition and behavior [32]. The dMPFC, therefore, may contribute to an increased variability of thought and conscious experience in schizophrenia.

#### Salience network and ventral attention network

Organisms are constantly exposed to a large amount of information originating from multiple sources. This makes it important that biologically relevant stimuli are quickly identified, including information related to rewards, threats, and ongoing tasks or goals [172, 279]. The salience and ventral attention networks (Fig. 1) are considered to support the early automatic identification of salient information originating exteroceptively, interoceptively, or within the thought stream [5, 20, 279–281]. Such initial automatic salience assessments allow important information to become amplified throughout neurocognitive hierarchies [279] through increased attention [282] and cognitive control [283]. The salience and ventral attentional networks, therefore, may underlie increased automatic constraints on thought. They may contribute to increased stability in thought and conscious experience when a singular piece of salient information is focused on but could also contribute to increased variability when attention flits between multiple salient percepts.

There is considerable overlap in the subregions and functions of the salience network and ventral attention network and there is a lack of consensus as to whether these two networks should be viewed as separable [5, 20, 284, 285] or as a single network [3, 280]. When treated as a single network, both “salience network” and “ventral attention network” have been used as designation labels. Of their multiple subregions, the anterior insula is commonly referred to under either designation label [172, 279, 280, 282], the ACC mentioned more frequently during discussion of the salience network [69, 172, 232, 279, 286], and the temporoparietal junction and inferior frontal gyrus mentioned more frequently during discussion of the ventral attention network [280, 282, 287–289]. While we acknowledge the significant overlap between the salience network and the ventral attention network, here we treat them as separable networks when discussing how their PFC nodes may contribute to mental state dynamics.

The salience network is composed of the anterior insula and the ACC (Fig. 1), especially the rostral anterior cingulate cortex (rACC) [5, 172], extending rostrally and somewhat ventrally along the anterior cingulate cortex (Fig. 2). The rACC is thought to support appraisals of viscero-sensory signals (afferent signals reflecting the internal state of the body [232]) based on self-referential and conceptual knowledge [170]. Neural activation in the rACC increases when attention is directed internally rather than externally, such as toward one’s own stream of thought [15, 112, 181, 215, 235] or one’s own subjective emotional feelings [290–292]. Activation in the rACC is also observed during visceral and somatic pain, as well as during hypoglycemia [290, 293–295], underscoring the rACC’s involvement in processing viscero-sensory signals. Furthermore, rACC activation is greater when attention is turned toward the subjectively experienced affective qualities of interoceptive sensations, such as their subjective unpleasantness, rather than less affective qualities such as their location [290]. Conversely, subjective relief (or attention away) from pain unpleasantness during opioid analgesia is accompanied by corresponding changes in rACC activation and its functional connectivity with the midbrain periaqueductal gray [293]. These results suggest that rACC recruitment may increase automatic constraints on thought by augmenting the extent to which viscero-sensory information is featured in ongoing thought. This may contribute to an increased stability of thought and conscious experience, if the meaning attributed to viscero-sensory signals is stable over time.

The rACC exhibits strong anatomical connections with brain regions involved in processing physiological signals, including the hypothalamus, the insula, and the periaqueductal gray [175, 227, 295, 296], as well as strong anatomical connections to two main DN_CORE_ regions, the anterior medial PFC cortex and the posterior cingulate cortex [167, 171, 174, 175]. The DN_CORE_ is closely linked to autobiographical self-processing [5, 20, 215], so that this connectivity may uniquely allow the rACC to integrate viscero-sensory signals with self-referential autobiographical knowledge and constructs [297–299] to support the consistent interpretation of viscero-sensory signals over time.

If the consistent interpretability of viscero-sensory signals were to be disrupted, however, it is possible that the rACC may contribute to increased variability in thought and conscious experience. Indeed, alexithymia—a condition featuring difficulties identifying, differentiating, and describing one’s feelings, as well as a heightened perceptual sensitivity to viscero-sensory signals [300–302]—is associated with altered rACC structure and functional connectivity with the default network core [303–308]. Alexithymic individuals exhibit smaller rACC volume [307], dampened rACC activation in response to emotional imagery [305], and reduced resting-state functional connectivity between the rACC and default network core [308]. It is thus possible that alterations to the rACC and its connectivity with the default network core may underlie the diminished interpretability of viscero-sensory signals associated with alexithymia: a disconnect between viscero-sensory processing and autobiographical self-knowledge may prevent visceral information from being consistently interpretable. This may lead to an increased sensitivity to viscero-sensory signals due to their being unpredictable and ambiguous. Indeed, heightened perceptual sensitivity to viscero-sensory signals has been found to mediate the relationship between comorbid alexithymia and anxiety [309], while the etiology of certain forms of anxiety may reflect a compensatory response to excessively high uncertainty in viscero-sensory signaling [310, 311]. In other words, when the rACC is less able to support consistent interpretations of viscero-sensory signals, individuals may develop rigid or highly stable neurocognitive states over their lifetime as found in cases of viscero-sensory driven anxiety [5, 310, 311].

The rACC may thus contribute to greater stability or variability of conscious experience, depending on the level of variability in viscero-sensory signals. For example, in a situation when a particular viscero-sensory signal is strong and enduring (e.g., unmedicated pain from injury), the rACC may contribute to constraining conscious experience by continuously capturing the salience of the perceived pain and making it more likely to be continuously represented in thought and conscious experience over time. But in situations where viscero-sensory channels are more variable, such as during states of comorbid alexithymia and anxiety [309], the rACC may contribute to increased variability in thought and conscious experience by letting a variety of viscero-sensory signals impinge upon experience.

The ventral attention network comprises the anterior insula, ventral extent of the temporoparietal junction, and inferior frontal gyrus (IFG; Fig. 1) [5]. The IFG (sometimes referred to as the ventral frontal cortex; Fig. 2) is the prefrontal node of the ventral attention network, extending over the posterior portion of the IFG as well as partially into the adjacent opercular region and the posterior middle frontal gyrus [312]. How the IFG contributes to thought and conscious experience has remained relatively elusive, perhaps due to its multiple network affiliations (Fig. 1) [5, 45, 280, 313, 314], its cytoarchitectural diversity [315, 316], and its relatively pronounced hemispheric specialization [317]. Although an influential early account linked the right IFG to the inhibition of motor responses and task-sets [318], subsequent experimental evidence have shown that it is recruited when important salient visual cues are detected, regardless of whether that detection is followed by the inhibition of a motor response, the generation of a motor response, or no response at all [319].

The involvement of right IFG in the detection of unattended and unexpected stimuli, and in triggering shifts of attention to them, seems to be specific to spatially localized stimuli (usually visually presented) in the external environment. Therefore, the right IFG, through its affiliation with the ventral attention network, appears to support automatic constraints for salience detection for information in the external environment. So, while the rACC appears to detect salient stimuli that are interoceptive in nature, reflecting salient changes in the internal environment, the right IFG may perform a similar function for stimuli that are spatially localized in the external environment.

The right IFG also may exerts automatic constraint on brain regions involved in motor control. Intracranial electrical recordings show the IFG to have downstream effects upon the primary motor cortex [320] and subthalamic nucleus of the basal ganglia [321]. These constraining influences may underlie its role in behavioral inhibition [313, 318] and in supporting associations between rules and their expected outcomes toward the deployment of goal-directed behaviors [119]. Across multiple tasks—including the Stroop, Wisconsin Card Sorting, and Go/No-Go tasks—the IFG is found to exhibit fMRI-adaptation to the repetition of specific rule-outcome pairs but not to the repetition of rules or outcomes in isolation; it also shows increased functional connectivity to brain areas associated with rule representation and reward processing [322]. This IFG activation appears invariant to the stimuli or actions involved in a rule as it has been associated with various goal-directed behaviors toward stimuli in the external environment [323–325].

Because the right IFG shows transient recruitment in response to salient changes in the external environment [312], it may contribute to an increased variability of thought and conscious experience in environments marked by frequent changes in the salience and relevance of spatially localized external stimuli. For example, when navigating intersections through busy urban traffic, one’s attention may frequently re-orient to detect salient changes in traffic lights, other vehicles on the road, pedestrians’ actions, and anything else that may be relevant. In such situations, the right IFG may constrain cortical motor regions to guide appropriate changes in driving behavior but in doing so facilitate frequent changes in visuospatial attention related to the fast-changing external environment.

The right IFG may also in some circumstances contribute to reducing the variability of conscious experience. This may occur, for example, when the external environment is stable and relatively invariable with respect to the relevance and salience of its constituents. In such circumstances—for example, driving on a quiet road along a familiar route—attention may become perceptually decoupled and turn toward the internal stream of thought, which may be relatively freely moving [5] and more variable over time than the current external environment. Experimental work has provided evidence for this through work that examines “attentional lapses”, or the turning of attention away from an external task at-hand and toward the internal and task-unrelated stream of thought [326]. In such contexts, recruitment of the right IFG predicts the reduction of an “attentional lapse” and a return of attention toward external task-related stimuli [327]. Therefore, similarly to the rACC, the right IFG may underlie automatic constraints that contribute to either decreased or increased stability in thought and conscious experience, depending on current contextual features. However, whereas the rACC appears to primarily reflect features of the internal (interoceptive and conceptual autobiographical narrative) environment, the right IFG may primarily reflect features of the external perceptual environment.

### Top-down beliefs as constraints

Beliefs (or predictions) serve to increase the brain’s processing efficiency by allowing for only the unpredicted portions of incoming information to be sent upwards along neurocognitive hierarchies [84]. In this way, beliefs likely contribute to an increased stability in thought and conscious experience by constraining how lower levels of the brain process information relative to expectations about what that information should be. REBUS proposes that the sense of self or “ego” can be thought of as a constellation of implicit beliefs (about oneself and the world), whose relaxation (i.e., ego-dissolution) is crucial to the increased variability of conscious experience engendered by psychedelic compounds [32]. “Egoic” beliefs normally constrain thought and conscious experience in subtle ways, biasing the brain to process information in relation to a self; indeed, normal walking consciousness is pervaded by an experientially inseparable sense of selfhood -- the sense of being a continuous “I” that is distinct from the rest of the world [328]. These self-related beliefs come in multiple forms too, including those about the body (e.g., ownership, boundaries, location in space) and those about the mind (e.g., autobiographical narrative, thought ownership) [66]. Importantly, this does not imply the existence of a self but rather that it is useful for the brain to infer the existence of a self when navigating the world [328].

Psychedelics disrupt the normative sense of self: one of their most consistent and profound effects is a subjectively experienced “ego-dissolution” [24, 25, 28, 31, 72, 329–332]—an attenuation of mental and bodily self-experience, including a reduced availability of autobiographical structures and a loss of bodily boundaries or felt ownership [66, 69]. This results in an increased variability of thought and conscious experience [95], because information from lower levels of the neurocognitive hierarchy is “liberated” from the constraining influence of top-down beliefs [32]. Within the PFC, alterations to neural activity in the DN_CORE_, salience network, and frontoparietal control network, as well as their connectivity to network nodes outside of the PFC, appear closely related to the process of ego-dissolution [23–27, 29, 30, 61, 99, 100].

For instance, functional connectivity between the aMPFC and posterior cingulate cortex of the DN_CORE_ significantly decrease following psilocybin and LSD administration [23, 24], while magnitude of decrease in aMPFC activation and blood flow correlated with the intensity of subjective effects for psilocybin [23]. These results suggest that reductions to aMPFC neural activation and functional connectivity are important neural correlates underlying the subjective effects of psychedelics. Interestingly, a recent experiment with psilocybin found that negatively-valenced, or anxious, experiences of ego-dissolution were primarily associated with increased concentrations of glutamate (an excitatory neurotransmitter) in the aMPFC [25]. These findings could be interpreted as implicating increased aMPFC metabolism in disruptions to the characteristically increased variability of conscious experience during psychedelic states. It is possible that this reflects attempts to reinstate an otherwise dwindling autobiographical self-narrative through the augmentation of self-referential schema.

However, the autobiographical sense of self is not the only aspect of self-experience that becomes weakened under psychedelics, and may not even be the first to undergo attenuation [66, 328]. Early clinical research on psychedelics suggests that the bodily sense of self—being an embodied entity with clear bodily boundaries and ownership—may be subdued before mental aspects of self-experience become attenuated (e.g., autobiographical selfhood) [333, 334]. Cognitive neuroscience findings support this observation, with regions supporting viscero-sensory processing like the rACC exhibiting decreased global functional connectivity at least 30 min before regions supporting autobiographical processing such as the aMPFC, under psilocybin [61]. This suggests that psychedelics disrupt bodily self-experience before mental self-experience. Altered rACC structure and function is also associated with the subjective effects of psychedelics: reduced cerebral blood flow to the rACC under psilocybin is associated with the reported intensity of subjective effects [23], cortical thickness of the rACC is predictive of the reported emotional intensity of experience under psilocybin [26], and increased functional connectivity within the salience network a day following ayahuasca administration, including portions of the rACC, is predictive of the intensity of reported alterations to bodily experience during its effects [27]. These findings suggest that the rACC may support implicit beliefs about the meaning of viscero-sensory signals, whose relaxation is crucial to the phenomenology of self-experience under psychedelics.

Furthermore, the early relaxation of bodily self-beliefs may play a causal role in the subsequent relaxation of mental self-beliefs. Given that the aMPFC occupies a relatively higher hierarchical position than the rACC along the principal gradient of functional brain organization [29, 30, 335], it is possible that mental self-beliefs may be unable to minimize the prediction error associated with increased viscero-sensory signaling that is normally compressed by lower-level bodily self-beliefs. This may lead to reductions in the confidence (i.e., precision-weighting) assigned to mental self-beliefs (i.e., self-referential schema), given their predictive obsoleteness. Increased bottom-up signaling from early sensory cortices may also contribute to this effect: N,N-DMT (a subtype of DMT) substantially increases bottom-up information flow similar to that of exogenous visual stimulation during eyes-closed rest [73], and the early visual cortex exhibits increased resting-state functional connectivity with much of brain under LSD [24]—an effect that occurs even before alterations to the rACC under psilocybin [61]. It is also possible that psychedelics sequentially disrupt different levels of the neurocognitive hierarchy because of a time-lag in their pharmacological action across the brain, rather than due to   time-dependent changes to informational signaling per se. Regardless, the dual-relaxation of mental and bodily self-beliefs will likely be associated with the deepest form of ego-dissolution, since decreased hierarchical differentiation of the aMPFC and rACC is correlated to the intensity of subjective ego-dissolution [29, 30].

In addition to relaxing mental and bodily beliefs about the self, psychedelics also reduce the capacity for goal-related beliefs to support an increased stability of thought and conscious experience [24, 29, 30, 100]. Within predictive coding, goals can be understood as beliefs (or predictions) about long-term outcomes that are supported by the control networks, but whose constraining influence (precision-weighting) is dependent upon motivational processes supported by regions outside of the control networks [336]. In this way, goal-related beliefs constrain lower levels of the neurocognitive hierarchy to process incoming information relative to the expectation of goal-completion.

Both control networks have been shown to exhibit a particularly high expression of serotonin 2A receptors [337]. LSD and psilocybin appear to engender an acute decrease in the control networks’ functional integrity alongside an increase in their communication with other networks [24, 100] (i.e., these networks become less modularly segregated from other brain networks). Because a number of other networks can be seen as hierarchically subordinate to the control networks along certain gradients of hierarchical brain organization [30], one could view this effect as a reduction in the hierarchical structure of global brain function under psychedelics, a key principle of the REBUS model [32]. Consistent with this, recent evidence has shown the reduction or flattening of a hierarchical gradient related to the control networks under LSD and psilocybin [29, 30], while the reduced probability of occupying a functional connectivity state anchored on the control networks under psilocybin predicts the intensity of subjective effects [99]. These results suggest that psychedelics reduce the control networks’ ability to constrain neurocognitive dynamics with goal-related beliefs, thereby contributing to an increased variability of thought and consious experience.

In summary, psychedelics seem to reduce the constraining influence that mental self-beliefs, bodily self-beliefs, and goal-related beliefs have over lower levels of the neurocognitive hierarchy. By relaxing these beliefs, psychedelics reduce the extent to which incoming information is processed in terms of a mental self, bodily self, and goals, ultimately contributing to an increased variability of thought and conscious experience. This is due to an increase in the amount of bottom-up information flow that is no longer constrained according to hierarchically superior beliefs (or predictions).

## Future research directions and conclusion

Recent theoretical advances through the DFT and REBUS frameworks have highlighted the importance understanding mental dynamics and the sources of variability and stability of mental states across time. Here we brought together these two frameworks and their predictions with what is known about the functions of different prefrontal subregions. The present discussion suggests a number of future directions for research that could give rise to an improved understanding of the dynamics of conscious experience and their clinical implications.

First, an important conclusion from the preceding discussion is that constraints that may occur at the neural level do not necessarily result in increased stability of conscious experience over time. As noted, for example, in the discussion of regions such as the dACC, rACC, and inferior frontal gyrus, the neural constraints these regions contribute to could result in either increased or decreased variability of mental states over time, depending on the stability or variability of the internal and external environments. Therefore, it would be beneficial for future experimental and theoretical work to elucidate the complex and contextually dependent relationship between constraints at the neural level and constraints at the level of consciously experienced mental states.

Second, regional differences in contributing to greater stability or variability in thought and conscious experience could be understood further by examining possible gradients of specialization and functional organization across adjacent cortical regions. For example, future work could examine the potential information processing gradient along the ventral-dorsal axis of the ACC. At the turn of the century, rostral portions of the ACC were proposed to underlie more emotional processes while dorsal areas were linked to more cognitive ones [338]. Recent accounts have echoed and refined this proposal, suggesting rostral areas to underlie appraisals of viscero-sensory signals in relation to one’s autobiographical self-concept and more dorsal portions to support goal-directed processes such as predicting action outcomes to guide action selection [170] or determining whether the energetic costs of attentional deployment, physical behavior, and encoding new information are worth their contributions to goal attainment [162]. More work is needed to evaluate the specifics of this potential gradient, and whether it overlaps with other gradients in the cingulate.

Third, to be able to make stronger regionally specific predictions regarding specific prefrontal subregions, neuroscientific work related to large-scale brain networks could benefit from more fine-grained and regionally specific analysis approaches. In particular, the field of psychedelics research could benefit from a more specific discrimination between different brain networks and regions. Neuroscientific work on psychedelics often makes use of whole-brain measurements [29, 30, 61, 73, 75, 77, 99, 339]. These approaches have and will continue to be highly beneficial for understanding the effects of psychedelics on global brain measures, but may obscure the more specialized contributions made by specific brain regions and networks to psychological phenomena. Methods with greater specificity, such as seed-based structural [26], functional [23, 24, 28, 60, 340, 341], and neurochemical [25] designs and analyses are sometimes undertaken but not yet common in the psychedelic literature. Adopting such approaches more frequently, alongside global brain measures, would substantially increase regionally specific understandings of brain function.

Fourth, alongside functional gradients of cortical organization [29, 30, 122, 335, 342], there may also be evolutionary gradients of cortical expansion that could hold insights as to the phylogenetic development of stability and variability in thought and conscious experience. For instance, brain maps of cortical expansion from macaque to human display a pattern of frontal, parietal and temporal lobe expansion [343] that overlaps closely with the whole-brain distribution of serotonin 2A receptors [337]. An important direction for future work would be to investigate the relationship between brain serotonin 2A receptor distribution and hierarchical gradients, while also considering how agonism at receptors in these specific sites of cortical expansion may differentially affect cognition and behavior.

Finally, existing knowledge and interventions for clinical conditions could be enhanced by paying greater attention to the stability or variability of thought and conscious experience that they feature. The DFT and REBUS model (along with the entropic brain hypothesis and hierarchical predictive coding) have already led to new assessment tools and interventions for various clinical conditions. These include a behavioral assessment for detecting early-stage Alzheimer’s disease based off the prevalence of spontaneous thoughts [344], a new model for psychedelic assisted therapy [345], a proposal to treat disorders of consciousness with psychedelics [346], along with many others [20, 71, 347–353]. By improving their specificity of predictions at the subregional level of brain function, the DFT and REBUS may be able to extend their utility for future clinical applications.

Overall, the dynamics of thought and conscious experience hold promise to significantly advance our scientific understanding of brain function and organization, along with their clinically significant alterations. A more complete understanding of those dynamics, however, will be impossible without appreciating their significance and impact at multiple levels of brain organization. Although it can be challenging to combine different levels of explanation, such as large-scale network analyses and regionally specific functional localizations of brain function, we hope that our present undertaking hints at the benefits of doing so and motivates further work that attempts similar multi-level analyses of neural and mental functions.
